# Harnessing *Aspergillus* and host M genes for sustainable phyllosphere microbiome engineering

**DOI:** 10.1007/s44297-025-00046-3

**Published:** 2025-03-04

**Authors:** Zhe Wang, Jiakang Yin, Kenichi Tsuda

**Affiliations:** National Key Laboratory of Agricultural Microbiology, Hubei Hongshan Laboratory, Hubei Key Laboratory of Plant Pathology, College of Plant Science and Technology, Huazhong Agricultural University, Wuhan, 430070 China

The phyllosphere microbiota plays a crucial role in maintaining plant health and enhancing disease resistance [1–5]. Recent studies have indicated that plants actively shape their microbial communities prior to stress and selectively recruit beneficial microbes in response to pathogen infection, thereby establishing an extended layer of plant immunity [6]. Despite accumulating evidence highlighting the critical role of metabolites as mediators in plant–microbe interactions [7], the mechanisms by which microbiota-derived signals promote host resistance remain poorly understood. In a recent study published in *Nature Microbiology*, Fan et al*.* identified 2,4-Di-tert-butylphenol (2,4-DTBP), a small molecule produced by the phyllosphere-associated fungus *Aspergillus*, which enhances resistance of rice to the fungal pathogen *Rhizoctonia solani* [8].

By sterilizing the leaves of three representative rice cultivars with varying resistance to *R. solani*, researchers demonstrated a loss of cultivar-specific resistance, underscoring the essential role of the native phyllosphere microbiota in disease registance. To identify key microbes, they performed phyllosphere microbiome sequencing in the absence of pathogen inoculation and discovered a positive correlation between *Aspergillus* abundance and resistance levels. Researchers subsequently hypothesized that specific *Aspergillus* metabolites underlie this protective effect. This hypothesis was supported when metabolites derived from *Aspergillus cvjetkovicii* were shown to suppress *R. solani* infection. Through metabolomic and bioassay-guided approaches, they identified 2,4-DTBP as the active metabolite responsible for *R. solani* suppression. Notably, this inhibitory effect extends beyond rice, providing protection to other crops, such as cucumber, maize, soybean, and tomato, even under field conditions, highlighting its broad-spectrum potential.

Subsequent transcriptomic analyses of *R. solani* treated with 2,4-DTBP revealed significant downregulation of *R. solani AMT1*, encoding an ammonium transporter. The overexpression of *RsAMT1* promoted hyphal growth, sclerotia formation, and pathogenicity, indicating that *AMT1* positively regulates *R. solani* pathogenicity. 2,4-DTBP was found to reduce the accumulation of reactive oxygen species (ROS) in *R. solani*. Conversely, H_2_O_2_ treatment upregulated *AMT1* expression in *R. solani* as well as in another fungal pathogen, *Fusarium fujikuroi*. These findings collectively suggest that *A. cvjetkovicii*-derived 2,4-DTBP suppresses *RsAMT1* expression by decreasing ROS levels, thereby dampening its pathogenicity.

The composition and structure of the plant microbiome are shaped by the plant genotype, developmental stage, and nutrient uptake, reflecting plant adaptations to various environments to impact traits such as disease resistance beyond innate immunity [9, 10]. This adaptive capacity has been attributed to microbiome-shaping (M) genes, denoting the genetic basis by which plants manipulate and reshape their associated microbiome [11, 12]. However, the specific genes involved in microbiome manipulation and the underlying mechanisms remain largely unexplored. Traditional approaches to enhance crop immunity have focused on disease resistance (R) genes, which typically encode receptor-like proteins that act as signal transduction switches by sensing isolate-specific pathogen effectors to initiate robust but pathogen race-specific resistance [13]. However, R-gene-driven defenses are typically associated with a growth-immunity tradeoff [14]. In contrast, the M gene leverages the modification of the microbiome that can provide direct pathogen resistance, thereby mitigating the potential growth-defense tradeoff associated with R genes. Consequently, M-gene-mediated strategies may offer a more sustainable solution for achieving broad-spectrum resistance without compromising crop yield. Future breeding programs could benefit from integrating M-gene-mediated microbiome shaping and R-gene-mediated resistance, combining the advantages of both approaches. Hence, understanding the interplay between M gene-mediated microbiome reshaping and microbiome-mediated disease resistance is vital for advancing disease management.

Nevertheless, our understanding of how microbiome-derived molecules influence plant–microbe interactions at the molecular level remains poorly understood. This study integrates genetic and chemical analyses to unveil the novel functional roles of the phyllosphere microbiota, offering promising avenues for harnessing microbial molecules in sustainable plant protection strategies (Fig. 1) [15]. Given the substantial diversity of the microbiota across different environments, future investigations should focus on how various microbial communities are manipulated by M genes (Fig. 2) and how these shifts translate into either broad-spectrum or species-/cultivar-specific resistance. Notably, as the cultivars examined in this study exhibit stable resistance under various field conditions, identifying the causal microbes and probing the functional redundancy orchestrated by the plant M gene should provide deeper insights into the genetic and ecological bases of microbiota-driven disease suppression for sustainable agriculture. Importantly, Fan et al*.* demonstrated that the M gene is dispensable for the beneficial function of *Aspergillus*. This means that *Aspergillus* can be utilized for crops irrespective of the presence of the M gene. Indeed, they showed that *Aspergillus* provides disease resistance to multiple crops, demonstrating broad applicability in disease management.

**Fig. 1:**
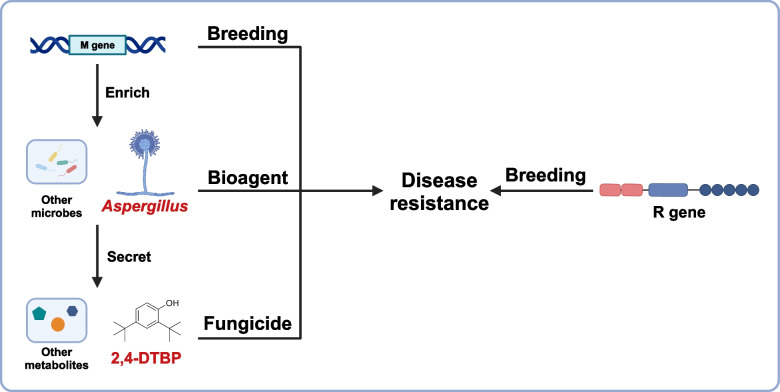
Integrated strategies for plant disease resistance: R-gene immunity, M-gene microbiome engineering, and microbial applications. This figure illustrates the multifaceted approaches to enhancing plant disease resistance. The R gene pathway (right) represents traditional breeding strategies to introduce resistance (R) genes into plants, conferring registance against pathogens, although this may come with a potential growth-defense tradeoff. The M gene pathway (left) highlights microbiome engineering, where the M gene enriches beneficial phyllosphere microbes, such as *Aspergillus cvjetkovicii*. These microbes secrete metabolites (e.g., 2,4-DTBP) that directly inhibit pathogens. Both strategies converge to bolster disease resistance. Additionally, bioagent applications (e.g., *Aspergillus*) and fungicide treatments (e.g., 2,4-DTBP) serve as complementary tools to reinforce plant defense mechanisms. Created in BioRender. Tsuda, K. (2025) https://BioRender.com/h74u688

**Fig. 2:**
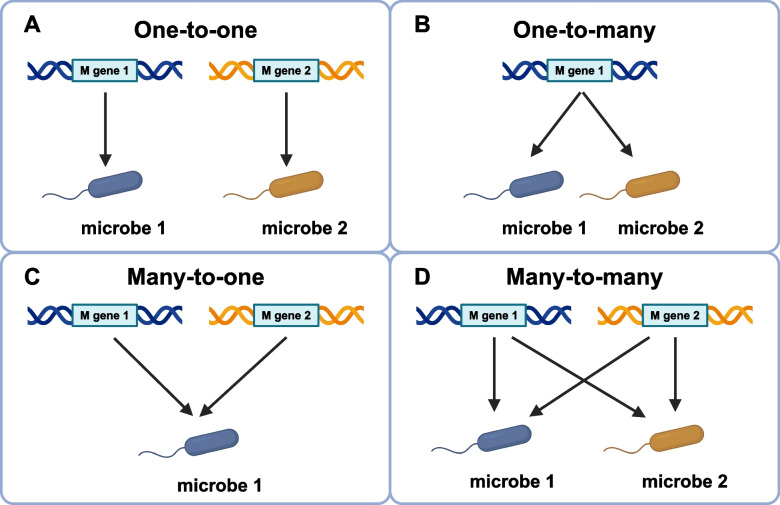
Hypothetical models of M gene-mediated plant microbiome manipulation. This figure presents a conceptual framework illustrating different modes of M gene regulation in shaping plant-associated microbiomes. The model is generalizable across plant species and tissues rather than being specific to any particular species or environmental condition. **A** Different M genes regulate specific microbial species in a one-to-one relationship, suggesting distinct genetic control over microbial interactions. **B** A single M gene regulates multiple microbial species, indicating a broad-spectrum regulatory function that manipulates various members of the microbiome. **C** Multiple M genes exert additive or redundant effects on a single microbial species, suggesting a redundant genetic network that ensures robust control over key microbial partners. **D** Multiple M genes interact with multiple microbial species, forming a complex regulatory network that coordinates the plant-microbiome interplay. Created in BioRender. Tsuda, K. (2025) https://BioRender.com/h74u688

## Data Availability

Not applicable.

## References

[1] Gong T, Xin X. Phyllosphere microbiota: community dynamics and its interaction with plant hosts. J Integr Plant Biol. 2021;63(2):297–304. 10.1111/jipb.13060. 33369158 10.1111/jipb.13060

[2] Vorholt JA. Microbial life in the phyllosphere. Nat Rev Microbiol. 2012;10(12):828–40. 10.1038/nrmicro2910. 23154261 10.1038/nrmicro2910

[3] Du Y, Han X, Tsuda K. Microbiome-mediated plant disease resistance: recent advances and future directions. J Gen Plant Pathol. 2024;91:1–17. 10.1007/s10327-024-01204-1.

[4] Vannier N, Agler M, Hacquard S. Microbiota-mediated disease resistance in plants. PLoS Pathog. 2019;15(6): e1007740. 10.1371/journal.ppat.1007740. 31194849 10.1371/journal.ppat.1007740 PMC6564022

[5] Liu X, Matsumoto H, Lv T, Zhan C, Fang H, Pan Q, et al. Phyllosphere microbiome induces host metabolic defence against rice false-smut disease. Nat Microbiol. 2023;8(8):1419–33. 10.1038/s41564-023-01379-x. 37142774 10.1038/s41564-023-01379-x

[6] Nakagami S, Wang Z, Han X, Tsuda K. Regulation of bacterial growth and behavior by host plant. Annu Rev Phytopathol. 2024;62:69–96. 10.1146/annurev-phyto-010824-023359. 38857544 10.1146/annurev-phyto-010824-023359

[7] Jacoby RP, Koprivova A, Kopriva S. Pinpointing secondary metabolites that shape the composition and function of the plant microbiome. J Exp Bot. 2021;72(1):57–69. 10.1093/jxb/eraa424. 32995888 10.1093/jxb/eraa424 PMC7816845

[8] Fan X, Matsumoto H, Xu H, Fang H, Pan Q, Lv T, et al. *Aspergillus cvjetkovicii* protects against phytopathogens through interspecies chemical signalling in the phyllosphere. Nat Microbiol. 2024;9(11):2862–76. 10.1038/s41564-024-01781-z. 39103572 10.1038/s41564-024-01781-z

[9] Sasse J, Martinoia E, Northen T. Feed your friends: do plant exudates shape the root microbiome? Trends Plant Sci. 2018;23(1):25–41. 10.1016/j.tplants.2017.09.003. 29050989 10.1016/j.tplants.2017.09.003

[10] Zhang J, Liu W, Bu J, Lin Y, Bai Y. Host genetics regulate the plant microbiome. Curr Opin Microbiol. 2023;72:102268. 10.1016/j.mib.2023.102268. 10.1016/j.mib.2023.102268 36708613

[11] Su P, Kang H, Peng Q, Wicaksono WA, Berg G, Liu Z, et al. Microbiome homeostasis on rice leaves is regulated by a precursor molecule of lignin biosynthesis. Nat Commun. 2024;15(1):1–16. 10.1038/s41467-023-44335-3. 38167850 10.1038/s41467-023-44335-3 PMC10762202

[12] Zhan C, Wang M. Disease resistance through m genes. Nat Plants. 2024;10(3):352–3. 10.1038/s41477-024-01644-9. 38409293 10.1038/s41477-024-01644-9

[13] Liu X, Ao K, Yao J, Zhang Y, Li X. Engineering plant disease resistance against biotrophic pathogens. Curr Opin Plant Biol. 2021;60:101987. 10.1016/j.pbi.2020.101987. 10.1016/j.pbi.2020.101987 33434797

[14] He Z, Webster S, He S. Growth-defense trade-offs in plants. Curr Biol. 2022;32(12):R634–9. 10.1016/j.cub.2022.04.070. 35728544 10.1016/j.cub.2022.04.070

[15] Zhan C, Matsumoto H, Liu Y, Wang M. Pathways to engineering the phyllosphere microbiome for sustainable crop production. Nat Food. 2022;3(12):997–1004. 10.1038/s43016-022-00636-2. 37118297 10.1038/s43016-022-00636-2

